# Polyethylene Terephthalate (PET) Bottle-to-Bottle Recycling for the Beverage Industry: A Review

**DOI:** 10.3390/polym14122366

**Published:** 2022-06-11

**Authors:** Patnarin Benyathiar, Pankaj Kumar, Gregory Carpenter, John Brace, Dharmendra K. Mishra

**Affiliations:** 1 Department of Food Technology, Mahidol University, Kanchanaburi Campus, Kanchanaburi 71150, Thailand; patnarin.ben@mahidol.ac.th; 2 Research & Development, Amcor Rigid Packaging, Manchester, MI 48158, USA; pankaj.kumar@amcor.com (P.K.); gregory.carpenter@amcor.com (G.C.); 3 The Whole Package LLC, Saline, MI 48176, USA; jgbrace@gmail.com; 4 Department of Food Science, Purdue University, West Lafayette, IN 47907, USA

**Keywords:** PCR, rPET, recycling process, sustainability, legal compliance, chemical recycling, advanced recycling, beverage, food contact surface

## Abstract

Disposal of plastic waste has become a widely discussed issue, due to the potential environmental impact of improper waste disposal. Polyethylene terephthalate (PET) packaging accounted for 44.7% of single-serve beverage packaging in the US in 2021, and 12% of global solid waste. A strategic solution is needed to manage plastic packaging solid waste. Major beverage manufacturers have pledged to reduce their environmental footprint by taking steps towards a sustainable future. The PET bottle has several properties that make it an environmentally friendly choice. The PET bottle has good barrier properties as its single-layer, mono-material composition allows it to be more easily recycled. Compared to glass, the PET bottle is lightweight and has a lower carbon footprint in production and transportation. With modern advancements to decontamination processes in the recycling of post-consumer recycled PET (rPET or PCR), it has become a safe material for reuse as beverage packaging. It has been 30 years since the FDA first began certifying PCR PET production processes as compliant for production of food contact PCR PET, for application within the United States. This article provides an overview of PET bottle-to-bottle recycling and guidance for beverage manufacturers looking to advance goals for sustainability.

## 1. Introduction

Plastic packaging accounts for 70% of the market of consumer products. Beverage packaging can primarily be classified into cold fill (such as aseptic), carbonated soft drinks, and hot fill. In choosing the appropriate beverage packaging material, the material must be able to withstand filling/handling temperatures, sustain the quality of the packaged beverage over its intended shelf life, and withstand internal pressurization requirements. Thermal stability requirements are dictated based on the operation for sterilization of the beverage and the container. Cold-fill beverages do not require sterilization. These will include water and high-acid beverages. Hot-fill beverages include high-acid and acidified products such as isotonic drinks, teas, and juice. Carbonated beverages (such as soft drinks) will require a package that does not deform with internal pressures up to 5 bar at room temperature. Choice of packaging material plays an important role in preventing degradation, in maintaining organoleptic qualities (scent, flavor, texture) and nutritional value (e.g., prevention of vitamin C oxidation in orange juice), and in ensuring consumer health and safety. Several packaging materials are appropriate in barrier and mechanical properties for use in beverage packaging. Beverage packaging includes glass bottles, aluminum cans, foil-laminated carton boxes, foil-laminated flexible pouches, and plastic bottles [1,2]. 

Polyethylene terephthalate (PET or PETE), which is a polyester plastic, is one of the most widely used packaging materials for beverages. Due to its excellent transparency, light weight, gas and water barrier properties, impact strength, UV resistance, and unbreakability (compared to a glass bottle), the production and use of PET bottles for beverage packaging has consistently increased worldwide. PET is a recyclable solution with performance benefits that are not available in alternative packaging options, such as glass bottles, aluminum cans, paperboard cartons, and other plastics. According to the data extracted from Euromonitor International (London, England), in the beverage industry, the PET bottle accounts for 67% of the market share between water, carbonated soft drinks, energy drinks, tea, and coffee. For single-serve bottles (<1 L), PET accounted for 44.7% of single-serve beverage packaging in the US in 2021. In comparison, aluminum cans accounted for 39%, glass for 11%, and high-density polyethylene (HDPE) for 3.4%. 

As with most plastics, PET is a petroleum-based polymer, and it does not readily decompose when released into earth’s environments via plastic waste leakage. In 2015, global plastic waste generated was approximately 141 million tons [3]. Packaging waste buried in landfills can still contribute to air, water, and soil pollution. Additionally, plastic leakage into landfills consumes available landfill space. It must be noted that the percentage of plastic in the landfill by volume is higher than that by weight [4]. Incineration of plastic packaging waste avoids consumption of landfill space and generates energy, but with the drawbacks of emissions creation and air pollution. The Great Pacific Garbage Patch is another example of unwanted plastic ending up in an undesirable place. Pollution from plastic waste, therefore, has been acknowledged as a major global environmental issue.

According to the United States Environmental Protection Agency (EPA) in 2018, 35.7 million tons of plastic waste was generated in the United States, which was 12.2% of total municipal solid waste (MSW). In addition to PET bottle waste, this plastic waste included polyolefin and polyester bags, wraps, bottles, and jars. Approximately 27 million tons of plastic (18.5% of US plastic waste) was discarded into landfills. Only 4.5% of plastic packaging was recycled [5]. According to European Economic Area data for the EU in 2019, 34.4 kg of plastic packaging waste was generated per EU inhabitant, on average. The EU recycled at a rate of 41% (14.1 kg) per inhabitant, on average [6]. Globally, PET accounted for 12% of total solid waste. The EU’s Commission for the Environment has conceived pathways by which member countries may significantly reduce plastic waste leakage. These pathways include concepts for incentivizing change in consumer behavior, improvement of waste management involving waste collection, waste separation and recycling, as well as the restriction of wastes accepted into landfills [7].

Source reduction, also known as waste prevention, is defined by the EPA as “a change in the design, manufacturing, purchasing or use of materials or products (including packaging) to reduce their amount or toxicity before they become municipal solid waste” [8]. To control and reduce plastic waste, source reduction can be implemented utilizing principles of waste management practice (reuse, reduce, redesign, and recycle packaging) in combination with packaging innovation. Many geographical regions, including North America, Europe, and South Asia, have encouraged a recycling program and/or adopted a policy for packaging waste management. These have included deposit systems, taxes, and plastic bag bans. 

Bottle reuse through returnable bottle systems has been widely practiced in South America and in several European countries [9,10,11], thus achieving waste reduction. However, in most regions, PET bottles are intended for single-use packaging and are disposed after first use. To reduce the environmental impact from packaging waste and to drive sustainability in plastic packaging, municipal recycling programs have been implemented to capture recyclable material streams. The inclusion of recycled plastics in food contact packaging has been slow to establish, due to preconceived concerns for food safety. However, with increased public pressures and increased adoption among manufacturers, this perception is beginning to change [12]. 

Post-consumer recycled (PCR) material can be used as the primary packaging material for food contact applications. As such, there has been a steady increase in the collection and recycling of PET bottles into PCR. PET recycling technology has been widely implemented and advanced for more than five decades [13,14,15]. These advancements continue with improved methods of recovery and conversion into PCR pellets. Moreover, bottle-to-bottle recycling development diverts this PCR pellet from downcycling applications. Considering the ensemble of bottle-to-bottle and bottle-to-strapping/textile/injection applications, PET has become one of the most successfully recycled plastic materials.

Processes for the recycling of PET packaging waste have become increasingly developed, but knowledge of the unit operations involved in these recycling processes has remained specific to recyclers. Beverage manufacturers harbor concerns regarding the food contact safety of PCR produced from PET containers that may have previously packaged chemicals or household products. Hazardous compounds potentially absorbed into the PET polymer may migrate into food stuff if they are not properly removed during recycling [16]. Available methods, such as the management of waste collection, super clean process, and advanced recycling, offer pathways to produce PCR PET with the removal of chemical contamination. These processes have the potential to produce PCR PET with contaminant levels similar to virgin PET [17]. 

The objective of this review article is to provide a framework for beverage manufacturers when making the decision on whether to consider PCR PET options for beverage products. The pertinent details about recycling, applications, food safety, regulations, and future trends are discussed in detail to assist these manufacturers. 

## 2. PET and the Processes for Post-Consumer Recycling

**Figure 1:**
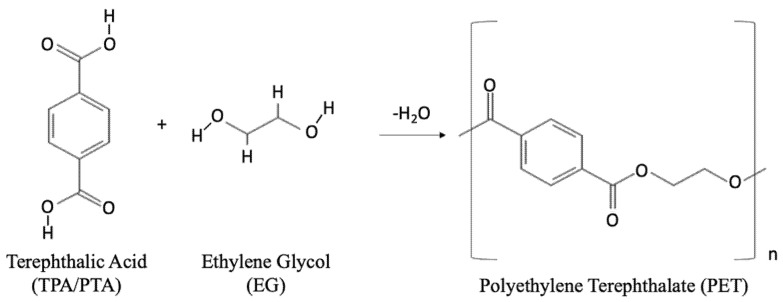
Chemical reaction of terephthalic acid and ethylene glycol for the formation of polyethylene terephthalate.

PET is a linear thermoplastic polyester made by condensation reaction between ethylene glycol (EG) and terephthalic acid (TPA), also called purified terephthalic acid (PTA), as shown Figure 1.

Both EG and TPA are most commonly derived from petroleum feedstock. PET may also be formed via transesterification of dimethyl terephthalate (DMT) with EG to form bis(2-hydroxyethyl) terephthalate (BHET), followed by reaction of the BHET with the extending PET chain (releasing EG). 

**Figure 2:**
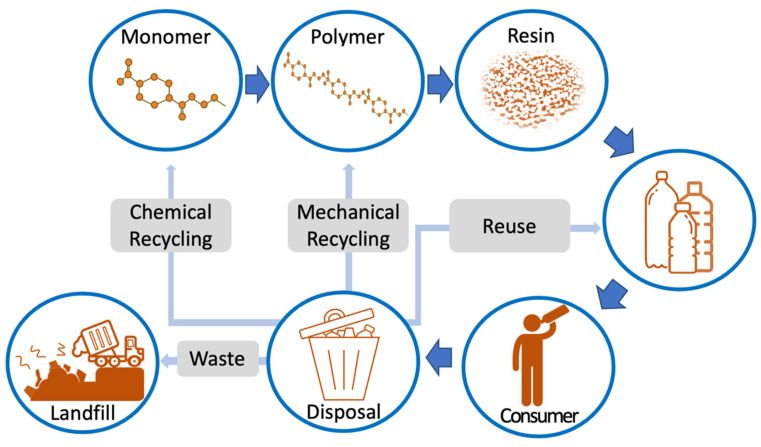
Plastic packaging life cycle of polyethylene terephthalate (PET) bottle.

Due to the condensation reaction, PET has non-carbon atoms in its main chain. The benzene component of the main chain imparts stiffness to the polymer. Depending on its processing and thermal treatment, PET may exist both as an amorphous and as a semi-crystalline polymer [18]. During thermal processing, BHET and low-molecular-weight polymer (oligomer) are additionally formed. PET has a glass-transition temperature (T_g_) between 67 °C (amorphous PET) and 81 °C (crystalline PET), and a melting point of ~256–260 °C [19]. The most common method for the production of PET bottles is two-step injection stretch blow molding (ISBM). Bottles are then filled and distributed to consumers. Following product consumption (when the bottles have completed their primary function), they are discarded and may enter various recycle streams. The plastic packaging life cycle below (Figure 2) shows a simplistic overview of the life cycle of the PET bottle.

### 2.1. Collection of PET Bottles

Proper disposal and collection are very important for plastic bottle recycling. Source separation, which is intended to remove the recyclable plastic before its leakage into landfill waste, can help to reduce contamination and improve recovery for recycling operations. Collection of post-consumer PET beverage bottles comprises two main systems: (1) curbside collection and (2) deposit system. 

For the curbside collection system, the PET bottles are segregated from non-PET recyclable materials into dedicated bales at the materials recovery facilities (MRF; also known as material reclamation facilities). MRFs are designed according to the local collection model. There are four models for curbside collection programs [20]: (1) Complete citizen separation, where consumers at home separate the waste into different recycle packaging material categories, such as paper, glass, metal, and plastic. Each of these streams are collected to a dedicated recovery facility. (2) Truck sorting, wherein following curbside collection, the driver is responsible for separating the materials into different categories, and further separation at the MRF is not required. An example of such a model is seen in La Mesa, California. (3) Site separation, where the entire separation occurs at the MRF, and there is no sorting done by either consumer or driver. The separation at the MRF facility is done by either manual or mechanical methods. The Rabanco recycling program in south Seattle is an example of this model. (4) Co-collection, where collection does not distinguish between normal trash and recycled materials. Such a mixed material stream poses significant challenges to the MRF. The cost of separation is also high in this case. Omaha, Nebraska has adopted this program.

The rules of operation for deposit systems are established via deposit-return legislation or bottle bills. Deposit systems use a monetary motivation to increase participation in recycling. These systems encourage recycling by adding a deposit fee to containers at the time the product is purchased from the retail store. Consumers receive the deposit back after returning the package to the collection center. The container deposit legislation (CDL), therefore, assists in establishing mono-material collection streams and, as a result, a better quality of PCR PET. In the US, ten states (California, Connecticut, Hawaii, Iowa, Maine, Massachusetts, Michigan, New York, Oregon, and Vermont) have adopted deposit laws. States with bottle bills have gained a beverage container recycling rate of approximately 60%, while recycling rates achieved in non-deposit states are closer to 24% [21]. Economic evaluations of the effectiveness of bottle bills have indicated that the higher the deposit fee, the greater the number of containers returned [22]. Bottle bills extensively increase the recycling rate for beverage containers and affect consumer behaviors. However, in certain US states, political resistance and beverage industry opposition pose challenges to enacting deposit systems [23]. Collection methods are significant in achieving contaminant reduction and ultimate quality of recycled PET [24,25]. Curbside and deposit collection programs overlap only partially. Curbside collection programs cover recyclable materials from the household, which are not covered by deposit programs. Deposit programs recover deposit containers from places that may not be served by curbside collection. The impetus of these collection programs is to minimize the solid waste problem and to provide avenues for the recovery of recyclable materials.

### 2.2. PET Value Recovery

Recycling technology has been developed as an alternative to traditional disposal into landfills. PET value can be recovered with four main methods: primary (re-extrusion), secondary (mechanical), tertiary (chemical), and quaternary (energy recovery) [26,27,28]. Each method provides its own advantages and disadvantages in terms of cost, quality, and environmental footprint. 

#### 2.2.1. Primary Recycling (Re-Extrusion)

Re-extrusion is a recovery process adopted at the converter (where plastic packaging is manufactured) to capture in-plant waste. Uncontaminated scrap is shredded into flake and mixed with virgin material or segregated as a second-grade (but uncontaminated) material for alternate molding uses [29]. Post-industrial recycled (PIR) materials are not considered to be PCR, as the material never reaches the consumer prior to its recovery [30].

#### 2.2.2. Secondary Recycling (Mechanical) 

Mechanical recycling is a conventional recycling method. After plastic containers are sorted and separated from associated contaminants, washed, and dried, they are ground (flaked), melted, and reprocessed into plastic pellets by extrusion. This process does not change the chemical nature of the polymeric material. Physical recycling does, however, impact the molecular weight of the recycled PET [31]. PET is a hydrophilic polymer and will attract moisture. Under high-temperature conditions (e.g., melt extrusion), reaction with water results in hydrolytic degradation (hydrolysis) and the reduction of average molecular weight (MW). Molecular weight reduction affects mechanical properties, melt viscosity, and impact resistance of the polymeric material [32]. To mitigate this issue, the PCR manufacturers usually increase the molecular weight of the PET by solid-state polymerization (SSP), where the polymer is heated below its melting point but above the glass transition temperature to remove the condensate byproducts, yielding a polymer with higher molecular weight. This process improves intrinsic viscosity (IV) and eliminates volatile organic compounds (VOC). Following SSP, recycled PET can be analyzed for benzene and limonene content. Benzene is a byproduct of PVC contamination and degradation. Limonene is a flavoring agent primarily carried over from residual citrus juices and may potentially impact the organoleptic properties of the packaged beverage [28]. Quantification of these components is indicative of the VOC removal effectiveness of the SSP, which in turn is indicative of a PCR being suitable for food contact application. Mechanical recycling of PET has been widely adopted in several countries [33]. 

#### 2.2.3. Tertiary Recycling (Chemical)

**Figure 3:**
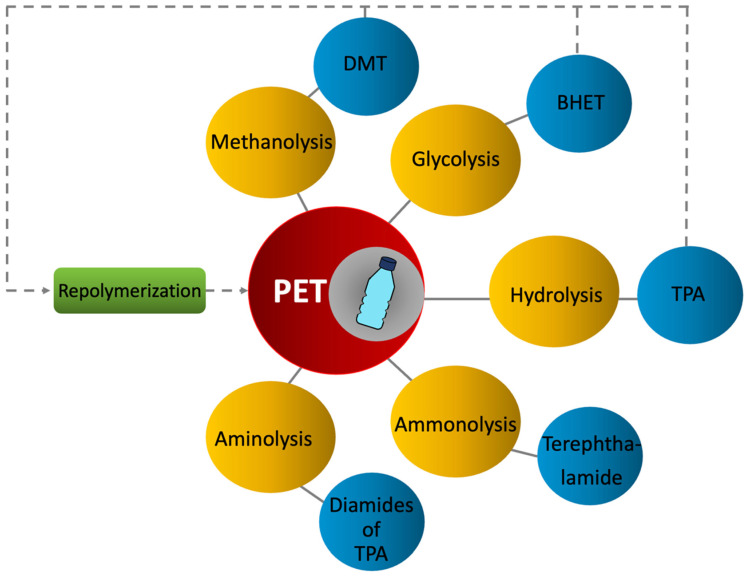
Chemical recycling of PET.

Chemical recycling (also known as advanced recycling) is a process by which the PET polymer is either depolymerized into its original components and repolymerized to a new oligomer or solvated (solvolysis) to dissolve the polymer for subsequent purification [34]. The polymer chain is, thus, either partially broken into smaller oligomers or fully broken down into monomer units, liquids, and gases [35]. The monomer can be refined for manufacturing by unit operations that are not suited to longer-chain polymers. Chemical recycling is suitable for heterogeneous materials or contaminated plastic containers, and it requires minimum pretreatment of the plastic waste. Because the material of interest is broken down into much smaller molecules, it is possible to use finer filtration and thus achieve much better purification of material than what is possible with mechanical recycling. This method can be used to recycle PET and other polymers such as polyamine, polyurethanes, and polyethylene [30]. Chemical recycling of PET has been actively studied in recent years [36,37]. PET can be chemically recycled in five different ways: methanolysis, glycolysis, hydrolysis, ammonolysis, and aminolysis. Only methanolysis and glycolysis, however, are primarily applied on a commercial scale [38]. Significant detail is given in this section (Section 2.2.3) because of the importance of reaction yields, the useability of particular monomers for subsequent PET re-polymerization, and the impact of chemical residual content on processing and final-article (PET bottle) properties. Figure 3 shows a summary of different PET chemical recycling methods and types of products. 

##### Methanolysis

**Figure 4:**
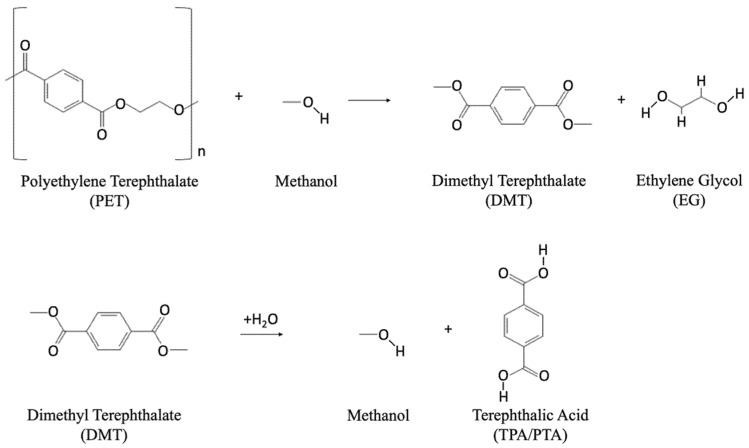
Chemical reaction for the methanolysis chemical recycling method.

PET can be depolymerized to DMT and EG by reaction with methanol under high pressures (2–4 MPa) and temperatures (180–280 °C) [39,40]. DMT may be repolymerized to PET following two reaction pathways: (1) transesterification of DMT with EG to form BHET (releasing methanol), followed by reaction of the BHET with the extending PET polymer chain to form longer-chain PET (releasing EG); and (2) hydrolysis of DMT to TPA and methanol, followed by reaction of the recovered TPA and EG. Catalysts utilized for methanolysis may include zinc acetate, magnesium acetate, lead dioxide, and cobalt acetate. The most common catalyst is zinc acetate [41,42]. During depolymerization, methanol is maintained in its liquid state via high-pressure reaction conditions. In addition to DMT and EG, reaction products may include a combination of alcohols, glycols, and phthalate derivatives [43]. DMT yield from methanolysis is in the range of 80–85%. Methanolysis follows the chemical reaction provided in Figure 4.

Research on the use of aluminum isopropoxide as a catalyst with a toluene and methanol mixture (20:80) has been reported to produce improved yields of monomer [44]. Following reaction, it is necessary to remove the catalyst to avoid future polymer degradation and possible loss of DMT. DMT is separated by centrifugation and crystallized to recover it from the postreaction mixture. Both batch and continuous processes can be utilized for the methanolysis process [38,40]. 

Supercritical methanol may also be utilized to depolymerize PET. Processing temperatures of 270–300 °C and pressures of 0.1–15 MPa are required for the depolymerization reaction. High-molecular-weight PET decomposes at a faster rate than lower molecular weight PET [45]. DMT is easier to purify than BHET produced by glycolysis. Methanolysis is, therefore, preferred for low-quality, low-cost feedstock, which does not compete with the mechanical recycling processes for feedstock. However, it is more costly and energy intensive than the glycolysis process [46,47]. 

##### Glycolysis

**Figure 5:**
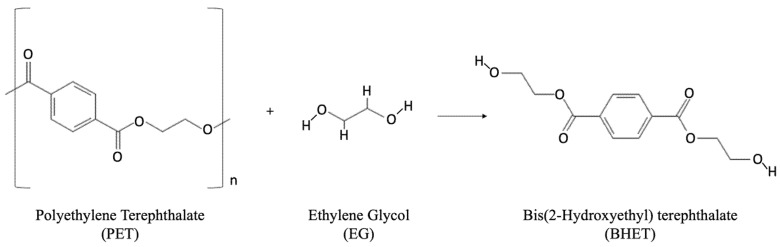
Glycolysis chemical reaction of PET with ethylene glycol to produce BHET.

Glycolysis is the most economical and commercially feasible method for chemically recycling PET. It is usually used for recycling of high-quality PET bottles, and it may compete with mechanical recycling for feedstock. Glycol (particularly EG) is used to decompose the ester linkages in the PET chain to produce BHET as shown in Figure 5. 

The reaction occurs under pressure. Temperatures required for reaction are in the range of 180–240 °C [28,38]. The glycolysis reaction is commonly performed in an extrusion process [47]. A catalyst is needed to enhance the reaction rate for BHET production. Glycolysis catalysis research has focused on: (1) development of highly efficient catalysts, (2) optimization of reaction conditions (time, temperature, PET/EG ratio), and (3) optimization of catalyst concentration, in order to maximize the reaction rate and the BHET yield [48,49,50,51,52,53,54,55]. 

Four main methods exist for the depolymerization of PET via glycolysis: catalyzed glycolysis, solvent-assisted glycolysis, supercritical glycolysis, and microwave-assisted glycolysis [56].

1. Catalyzed glycolysis

Several studies have reported zinc as providing optimal catalyst performance, as compared to transition metal catalysts. Some metal salts, especially zinc salts, are catalytically active at temperatures below 245 °C [57]. Zinc acetate, used as a catalyst in PET glycolysis, has been reported to produce the highest recovery of BHET monomer, as compared to other metal acetates such as lead, cobalt, and manganese [42,58]. Manganese acetate, as the glycolysis catalyst, has been demonstrated to react optimally in glycolysis under reaction conditions of 190 °C for 1.5 h with a concentration of 0.025 moles of manganese acetate per kg of recycled PET [59]. The glycolysis reaction rate dependently increases with increased reaction temperature and increased concentration of manganese acetate. Metal chloride catalysts such as zinc, lithium, didymium, magnesium, and iron can also catalyze glycolysis of PET bottle waste with performance equivalent to acetate salts. Zinc chloride catalyst at 0.5 wt%, with a PET:EG ratio of 1:14, also achieved BHET yields of 73.24% with 8 h of reaction time [60]. 

Heavy metals, such as zinc and lead, pose threats to the environment and require a controlled process for disposal. Mild alkalis, such as sodium carbonate and sodium bicarbonate, were proposed as glycolysis catalysts with fewer risks associated with handling and disposal [61]. Studies have shown that qualitative yields of BHET monomer from glycolysis reaction with sodium carbonate (61.5%) and sodium bicarbonate (61.94%) catalysts were comparable with zinc acetate (62.51%) and lead acetate (61.65%) catalysts. Moreover, other environmentally friendly catalysts including glacial acetic acid, lithium hydroxide, sodium sulfate, and potassium sulfate have also demonstrated yields of BHET comparable to conventional heavy metal catalysts [62]. 

Titanium–phosphate, when used as a glycolysis catalyst at 200 °C for 2.5 h with a concentration of 0.003 catalyst/PET by weight, has more recently been found to depolymerize PET with 97.5% BHET recovery. Catalytic activity is higher than that of zinc acetate, which in comparison has a 62.8% BHET recovery [63]. Three solid catalysts that have been evaluated for their catalytic ability in glycolysis include SO_4_^2−^/ZnO, SO_4_^2−^/TiO_2_, and SO_4_^2−^/ZnO-TiO_2_. All three materials demonstrated thermostability, superacid properties, and high catalytic activity. The SO_4_^2−^/ZnO-TiO_2_ catalyst produced 100% conversion of PET with 72% selectivity of BHET, after 3 h at 180 °C. Advantages of the solid catalysts include ease of removal by filtration and being non-corrosive; however, the reaction process requires high temperatures and pressures [52]. 

Although the catalysts are essential for effective glycolytic depolymerization of PET, non-solid catalysts are difficult to remove from the product and cannot be reused [64]. Effective removal is important to BHET purity. There is continued interest in the development of effective reusable catalysts for the depolymerization of PET waste into high-quality BHET monomer. These reusable catalysts include nanocomposites and ionic liquids. Metal oxide has been studied at the nano scale as a catalyst with intrinsic catalytic properties. Metal oxides are viewed as favorable in production due to their surface area and number of active sites [65,66,67,68,69]. Polyoxometalates (POMs) are inorganic metal-oxygen clusters, classified as nanomaterials. In the field of catalysis, POMs have been used in polymerization, decomposition, alkylation, transesterification, and esterification [70,71,72]. The study of transition-metal-substituted POMs K_6_SiW_11_MO_39_(H_2_O) (M = Zn^2+^, Mn^2+^, Co^2+^, Cu^2+^, Ni^2+^) as catalysts in the glycolysis of PET showed that SiW_11_Zn had the highest catalytic activity [73]. The reaction rate was fast (30 min) under mild conditions (atmospheric pressure at temperature of 185 °C), and the BHET yield was more than 84%. Depolymerization of PET can be achieved at a low catalyst/PET molar ratio (0.13%), with a PET/EG weight ratio of 1:4. POM catalysts can be separated from the BHET product by filtration. 

Ionic liquid (IL) is a liquid-form salt with a melt temperature below 100 °C. Ionic liquids were first studied for the purification of glycolysis products [49]. Ionic liquid has been studied as the primary glycolysis depolymerization catalyst. As a glycolysis catalyst, 1-butyl-3-methylimidazolium bromide ([bmim] Br) has been shown to achieve 100% conversion of PET following 8 h of reaction at atmospheric pressure at 180 °C. In addition, the catalyst could be used repeatedly. Additional ionic liquid catalysts evaluated for use with glycolysis depolymerization have included 1-butyl-3-methylimidazolium bicarbonate ([Bmim]HCO₃), 1-butyl-3-methylimidazolium chloride ([Bmim]Cl), 1-butyl-3- methylimidazolium tetra-chloroferrate ([Bmim]-FeCl_4_), 1-butyl-3-methylimidazolium hydroxyl ([Bmim]OH), 1-allyl-3-methylimidazolium halometallate, and 1-butyl-3-methylimidazolium acetate [56,74,75,76].

2. Solvent-Assisted Glycolysis

In solvent-assisted PET glycolysis, an organic solvent is utilized as a reaction accelerant. It has been demonstrated that a xylene solvent increases the catalytic ability of zinc acetate, and a BHET yield of 80% can be achieved. This yield is greater than what would be achievable without the xylene’s presence, due to the increase in miscibility of the PET-glycol mixture [77]. Since release of organic solvents is harmful to the environment, massive use of organic solvent for miscibility enhancement is not recommended [48].

3. Supercritical Glycolysis

Supercritical temperature and pressure conditions have been demonstrated to improve yield in PET hydrolysis, methanolysis, and glycolysis processes. Temperature and pressure are increased above the critical point for EG. With EG under supercritical conditions of 450 °C and 15.3 MPa, glycolysis yields of 93.5% BHET within 30 min have been demonstrated [78]. The shortened reaction time is due to the high solvent density, solubility, kinetic energy, diffusion rate, and reaction rate of supercritical ethylene glycol. The main advantage of this method is that no catalyst is required. Therefore, the difficulty in the separation of the catalysts from the reaction products is avoided. The main drawback is the energy required to achieve the temperatures and pressures required for the process [79]. The process of supercritical glycolysis provides a high yield of BHET with negligible yield of BHET dimer, diethylene glycol (DEG), and triethylene glycol (TEG) side products.

4. Microwave-assisted glycolysis

In microwave-assisted glycolysis, microwave radiation is utilized as the energy source for the depolymerization reaction. Microwave radiation activation has been demonstrated to reduce the glycolytic depolymerization reaction time from 8 h to 35 min. In contrast to supercritical glycolysis, BHET yield does not increase with the reduction in reaction time without the addition of a catalyst [80]. The use of alkali catalysts with microwave radiation for non-aqueous glycolysis has been shown to reduce the reaction time to less than 3 min. Further reduction in reaction time is a benefit to both energy and production efficiency. Alkali concentration and radiation time are key points for optimization due to their implications regarding process efficiency [81]. The use of zinc acetate as a catalyst for PET microwave-assisted glycolysis (0.5%, *w/w*) has likewise been demonstrated to minimize reaction time (30 min; much faster than the 8–9 h required for the conventional glycolytic process) [82]. Combination of ionic liquid catalysts with microwave radiation at 170–175 °C has been shown to produce 64% BHET yield, with PET conversion of up to 100% [83]. BHET yield may be further increased with a second glycolysis step to depolymerize residual oligomers. Glycol ratio, reaction time, and glycerol concentration are all important factors in maximizing production yield.

##### Hydrolysis

**Figure 6:**
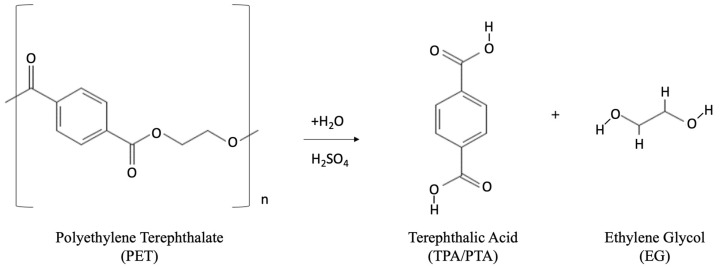
Chemical reaction for acid hydrolysis process.

PET can be depolymerized into EG and TPA by hydrolysis. Hydrolysis is not commonly used for recycled food-grade PET as it is costly to purify the recycled TPA. This process can be carried out using high pressures of 1.4–2 MPa and high temperatures of 200–250 °C under acidic, basic, or neutral conditions. The three main hydrolysis methods are as follows:

(1) Acid hydrolysis: Several acid substances can be employed in the process of acid hydrolysis, including concentrated sulfuric acid (H_2_SO_4_) (which is most commonly used), nitric acid (HNO_3_), and phosphoric acid (H_3_PO_4_). The use of concentrated sulfuric acid can help to reduce the energy consumption of the process via lowering of the requirement of high pressure and temperature in the reaction vessel [84,85]. TPA can be recovered from the PET feed scrap within several minutes under operating conditions of 60–93 °C in 87 wt% H_2_SO_4_ solution and 85–90 °C in 90 wt% H_2_SO_4_ solution [86]. The chemical reaction for the acid hydrolysis is shown in Figure 6.

Longer reactor residence times (several days) and high temperatures (100 °C) are required if the H_2_SO_4_ concentration is reduced to less than 80 wt%. EG is recovered from the final filtrate through extraction with suitable organic solvents, such as trichloroethylene [87]. It is, however, a very expensive method, and a large quantity of inorganic salts and aqueous waste are generated due to the highly corrosive nature of the chemicals.

**Figure 7:**
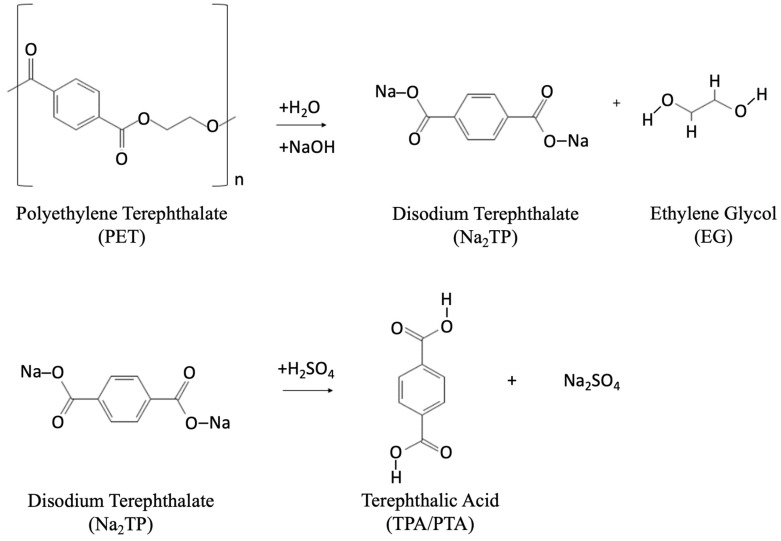
Alkaline hydrolysis chemical reaction to produce TPA.

(2) Alkaline hydrolysis: For the alkaline hydrolysis process (Figure 7), a 4–20 wt% aqueous alkaline solution of sodium hydroxide (NaOH) or potassium hydroxide (KOH) is used [38]. The process is conducted under a pressure of 1.4–2 MPa and at temperatures of 210–250 °C for 3–5 h. This reaction results in disodium or dipotassium terephthalate, which is further treated with sulfuric acid to separate TPA [88].

This process has potential for commercial-scale recycling as it can handle contamination well and still produce high-quality TPA. The yield of TPA directly depends on the reaction temperature and on the catalyst concentration [89]. Use of aqueous ammonia solution at 200 °C has also been shown to provide good results for PET alkaline hydrolysis [90]. Phosphonium quaternary salts have been shown to increase the reaction rate of alkaline hydrolysis at low operation temperatures (130–190 times greater) [54].

**Figure 8:**
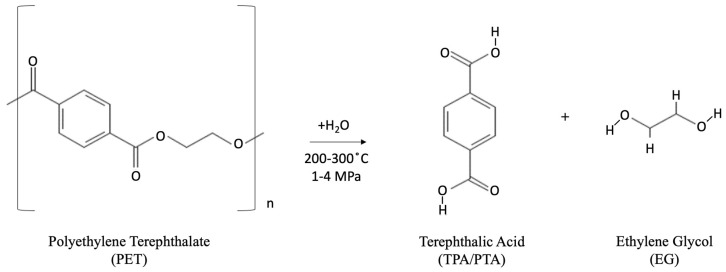
Chemical reaction during neutral hydrolysis process.

(3) Neutral hydrolysis: Hot water or steam at temperatures of 200–300 °C is used for the neutral hydrolysis reaction, with pressures of 1–4 MPa mainly used in this process. This hydrolysis method has been shown to produce high-purity TPA and EG monomers [46,91] and high yield at temperatures greater than 250 °C [92]. As with alkaline hydrolysis, the yield of TPA and EG directly correlates with increases in reaction temperature. The yield of TPA has been shown to reach 90% at reaction temperatures of 420 °C. The chemical reactions taking place during the neutral hydrolysis process are shown in Figure 8.

A maximum EG recovery up to 60% has been demonstrated when operating at a temperature of 300 °C. The catalytic dehydroxylation of EG might cause the lower yield of EG. During hydrolysis, diluted EG produced can be recovered through extraction or by distillation. With less formation of inorganic salts, compared to acid and alkaline hydrolysis, neutral hydrolysis can be considered a more environmentally friendly method. A drawback to this method is that the purity of TPA produced from neutral hydrolysis is lower than that of acid and alkaline hydrolysis, resulting in impurities in the repolymerized PET. The potential product contamination can be removed by filtration of TPA solution, which is dissolved in caprolactam or in sodium hydroxide solution [93]. The crystallization of TPA from caprolactam has been shown to produce TPA with a purity over 99% [94]. 

##### Ammonolysis 

**Figure 9:**
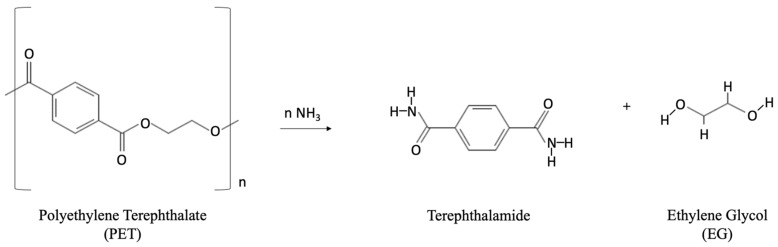
Chemical reaction during ammonolysis process to produce terepthalamide.

Ammonolysis achieves PET depolymerization via reaction of anhydrous ammonia (NH_3_) with PET in the presence of EG to produce terephthalamide (Figure 9). 

Terephthalamide may then be converted to terephthalonitrile, para-xylylene diamine, and/or 1,4-bis(aminomethyl)- cyclohexane [95]. Reaction temperatures and pressures of 120–180 °C and 2 MPa for 1–7 h are required for the depolymerization of post-consumer PET bottles [38,96]. At the end of the reaction, the amide is filtered, rinsed with water, and dried. The achievable product purity has been reported as greater than 99%, with the final yield exceeding 90% [95]. Low-pressure ammonolysis PET depolymerization, catalyzed by 0.05 wt% zinc acetate, has been demonstrated at a process temperature of 70 °C with a PET-to-ammonia ratio of 1:6. TPA amide generated in this study was reported with a yield of 87% [38]. 

##### Aminolysis

**Figure 10:**
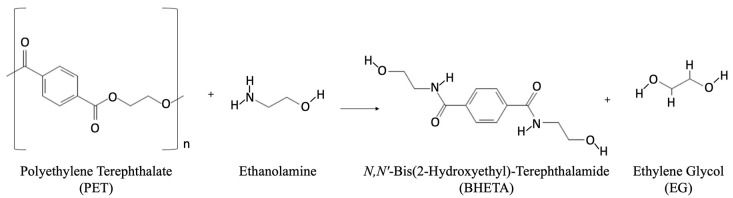
Aminolysis process chemical reaction for depolymerization of PET.

Aminolysis of PET generates diamides of TPA and EG. Commercially, this method has been less explored for PET recycling. Aminolysis is more commonly utilized to improve PET properties to produce PET fibers when processed in the forms of fibers and powder [97]. Aqueous amine solutions that are used to depolymerize PET in the aminolysis process include methylamine (the most commonly used), ethylamine (shown in Figure 10 chemical reaction), and ethanolamine at temperatures of 20–100 °C [97,98,99,100,101]. 

Other amines utilized for aminolysis include butylamine, triethylenetetramine, allylamine, polyamines, morpholine, and hydrazine [102,103,104,105]. Depolymerization of PET through aminolysis has been demonstrated using ethanolamine in the presence of catalysts such as glacial acetic acid, sodium acetate, and potassium sulfate. Pure form bis(2-hydroxy ethylene) terephthalamide (BHETA) was produced with 91% yield [106]. Microwave-assisted aminolysis, with the use of ethanolamine, demonstrated a high yield of BHET (nearly 90%) within a reaction time of 4 min [80]. 

A combination of chemical recycling processes can be used to enhance the depolymerization process and more effectively remove initial contamination from the waste PET [107]. Combined chemical methods include glycolysis-hydrolysis, methanolysis-hydrolysis, glycolysis-methanolysis, and glycolysis-aminolysis. The high purity potential of recycled PET (rPET) is what makes this product highly attractive for commercial applications. For the sustainable reuse of PET, chemical recycling makes use of raw material that has already been extracted from the environment. Consumption of our natural resources is reduced with the increased ability to recycle PET waste. Chemical recycling offers pathways to reduce the additional environmental surcharge and resources (monomer) required for PET production [48]. Chemical recycling can be an expensive method for PET recovery. In order to increase the interest and the adoption of chemical recycling, regulations such as carbon dioxide (CO_2_) emissions penalizations or other forms of taxation related to the regulation of environmental impact may be needed [108].

#### 2.2.4. Quaternary (Energy Recovery)

In this last method of drawing value from plastic waste, the energy content of the plastic is partially recovered. Conversion of the plastic into energy is accomplished via incineration in a furnace. Thus, chemical energy is converted into thermal energy. This process is suitable when other methods of separation and recovery are not suitable, due to heavy contamination. Energy produced from incineration can be converted into electricity and residuals from the incinerator can be safely disposed in landfills [28]. This controlled burning in the presence of air converts the waste feed into carbon dioxide and water. It is not possible to have zero emissions from the incinerators. In the US, incinerator emissions of particulate matter, carbon monoxide, dioxins/furans, sulfur dioxide, nitrogen oxides, hydrogen chloride, lead, mercury, and cadmium are regulated at the federal level by the Clean Air Act [109]. States work together with the EPA to ensure incineration facilities for municipal solid waste meet federal Maximum Achievable Control Technology (MACT) standards. 

## 3. Recycling Operation for PCR PET

### 3.1. Sortation and Purification 

Virgin PET is designed to purpose. Grades target specific ranges for intrinsic viscosity and crystallization rate, depending on the application (e.g., water, hot fill, carbonated soft drink, thermoforming). Tailoring of the virgin material is done with control over the input comonomer, comonomer content, and catalyst system. Additive packages are added at the resin manufacturer or converter to convey performance advantages. Additive packages may include, but are not limited to, reheat additives, toners, colorants, and oxygen barriers.

The PET recycle stream is initially composed of PET containers, container closures, labels, and residues from consumer products. As such, the recycle stream has the potential to contain high-density polyethylene (HDPE) and polypropylene (PP) from the closure. Adhesive, polyethylene terephthalate glycol (PETG), paper, and polyvinyl chloride (PVC) may enter the final recyclate from container labeling. In addition to product residues retained by the packaging, dirt and non-intentionally added substances may enter the recyclate from the reclamation process. 

**Figure 11:**
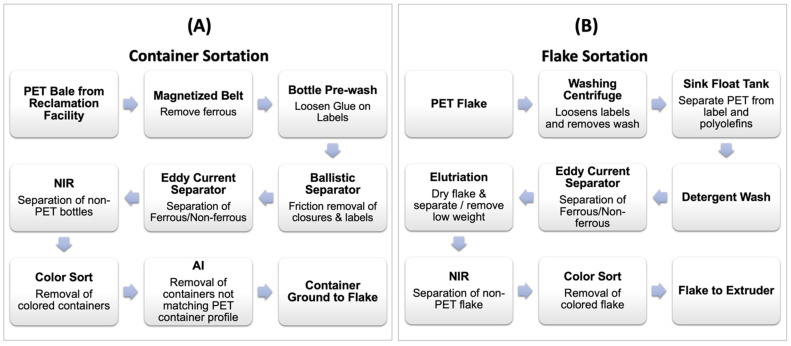
Unit operations in recycler: (**A**) container sortation and (**B**) flake sortation.

rPETs produced from mechanical recycling processes contain a portion of the contaminants entering the recycle stream. Contamination is reduced via various sorting unit operations, as shown in Figure 11A,B. These unit operations may include near-infrared (NIR) material identification and separation, color recognition and separation, eddy-current separation, float/sink density separations, and elutriation removal of labels. More recently, artificial intelligence (AI) has been implemented in the sortation process. Through deep learning and vision systems, AI can identify and pick non-PET containers to remove them from the recycle stream.

**Figure 12:**
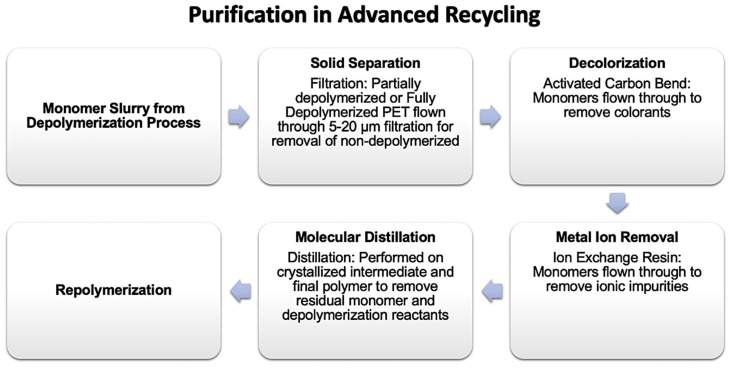
Unit operations in advanced recycling purification.

Advanced or chemical recycling of PET, described earlier, reduces the recycle stream to much smaller molecules, or even monomer blocks. Non-PET contamination is removed, and the repolymerized PET crystallization rate and IV can be tailored to the intended application, as shown in Figure 12. Management of process residuals from chemical recycling is non-trivial since these residues, particularly DEG, can affect both the copolymer composition (as co-monomer) and the flexibility of the re-polymerized PET (as internal slip agent).

IV in mechanically recycled PET is targeted via polycondensation reactions. The two main approaches to targeting IV are: (1) enhancing IV on the incoming PET flake, and (2) enhancing IV in the outgoing PET pellet. Both techniques make use of holding the PET in inert environments at a temperature of 190–210 °C. When IV is increased in the flake entering the extruder (e.g., Erema^®^ systems; chain extenders, solid-phase reactor polycondensation) flake is simultaneously dried (reducing hydrolysis and IV losses in the extruder). When increasing IV in the flake, volatiles are removed from the flake; however, volatiles generated during degradation in extrusion will remain with the finished product. Solid-state polymerization (SSP) is performed on the pellet outside of the extruder. SSP has the added benefit of volatiles removal for volatiles present before and generated during the extrusion process. For instance, it can lower the acetaldehyde and ethylene glycol, and hence minimize concentration of 2-methyl-1,3-dioxolane [110].

Mechanical recycling of PET with an appropriate level of repolymerization can produce a product with IV suited to purpose. The contamination extent of non-additives in the final mechanically recycled product is dependent on the efficacy of the sortation operations. Even in the case of highly efficient non-additive removal, the final mechanically recycled rPET will contain a blend of the various comonomers, catalysts, toners, colorants, and functional additives present in the incoming stream. 

### 3.2. Super Cleaning Process

Non-food contamination poses a risk to consumer safety [25]. Non-food PET bottles, which may have previously contained solvents or harmful chemicals, are inevitably collected in the PET recycle stream and fed into the recycle process. Non-food containers, thus, can contaminate the rPET product. It is desirable to keep these non-food PET bottles in the collection stream. Super-clean recycling, also called deep-clean recycling, has been used to increase the efficient decontamination of recycled PET bottles for re-use in direct food contact packaging. There are three stages to the super-clean process: (1) high-temperature wash, (2) gas wash, and (3) chemical wash [16,111]. More specifically, the super-clean process makes use of high temperature washing, vacuum, surface treatment, melt filtration, and melt degassing to remove contamination [112]. In the flake wash, chemicals such as caustic soda (with the assistance of ethylene glycol) hasten the contaminant removal. SSP is likewise employed to remove contaminants. Heating the PET to 200 °C brings the contaminants to the surface of the PET pellets or flakes. Next, vacuum or inert gas treatment is applied to mobilize and remove the contaminants from the surface and out of the recyclate stream. 

Cleaning efficiency depends on the quantity of non-food bottles in the recycling feed stream. Super-clean bottle-to-bottle recycling was first developed at plant scale in Beaune, France [16]. Several countries, including the US, Netherlands, Germany, Switzerland, and Australia, have followed with the construction of super-clean plants [113,114].

## 4. Processing and Performance Differences between Virgin PET and Post-Consumer Recycled PET

PCR PET behaves differently than virgin PET in several different ways. The process required to make a container with a given grade of PCR is not just a material change. Processing could be challenging for individuals not skilled in the art. When manufacturing containers with virgin PET, the process tends to be repeatable every time new production is started. When using PCR, different lots from the same supplier can behave very differently. This is because the ingredients or raw materials that go into producing the PCR can vary from batch to batch. For beverage containers, the virgin material primarily falls in three categories:

(1) Water grade (low IV, acetaldehyde suppression, could have additives to enable ultrathin bottle walls);
(2) Heat-set grade (higher IV, DEG suppression, co-monomers to suppress hot-fill shrinkage, could have additives to aid reheating and crystallization);
(3) Carbonated soft drink (CSD) grade (highest IV, co-monomers to resist expansion).

When bottles made from these resins are recycled, each bale of bottles may contain different levels of the described virgin grades. The final PCR PET pellet may have many different co-monomers, additives, and levels of additives. Additionally, depending on impurities and colors included in the feed stock, the resultant color of PCR may vary. PCR manufacturers try to minimize these influences, but the converter needs to plan and adapt to variation. Variability is pronounced due to seasonality (as consumption patterns change with the season) and bottle bale source (when including various geographies feeding into the recycled stream). Variability in PCR material properties impact converter processing and the functionality of the product. Converter risk includes impact to preform injection stability and to the stability of the blow molding processes. Variation in PCR may impact the consumer’s sensory experience of the product, both visually and during consumption. The bottle manufacturing process can impact the final PCR PET bottle quality.

### 4.1. Injection

#### 4.1.1. Drying of Material and Injection Pressure

PET requires drying before processing to avoid loss of molecular weight or intrinsic viscosity (IV). During storage, PCR tends to pick up moisture faster compared to virgin material. This difference in moisture adsorption/absorption may be due to differences in crystallinity between the virgin and PCR plastic pellets. Lower crystallinity results in easier propagation of moisture into the plastic pellet from the atmosphere. PCR can be lower in crystallinity compared to virgin material, and the shell of the pellet can be less crystalline than its core. During injection of preforms, the crystalline shell melts, but not the higher crystallinity core. The screw recovery and barrel temperature profile will need to be adjusted to avoid gumming issues. During the recycling process, vinyl ester end groups can generate, and they can cause yellowing or browning of PET during drying [115]. As a result, some PCRs require drying at relatively low temperatures for longer durations. Longer dryer times pose a productivity issue. Differences in dryer time requirements between PCR and virgin PET present a significant operational challenge when drying a pellet blend. A balance needs to be struck to ensure both materials are dry without degradation. With lot-to-lot variation in PCR, chosen conditions need to be applicable to a range of material properties. 

#### 4.1.2. AA Generation

Acetaldehyde (AA) is a known byproduct of processing PET. It is a naturally occurring flavoring compound, which is perceived as a sweetener. AA is typically a problem with unflavored water, where the consumer expects odorless and flavorless product. With PCR composition varying lot-to-lot, the risk of AA generation can change from lot to lot. While this may not be a critical concern if the PCR is being used for hot-fill or carbonated sweetened products, this is critical to the injection of preforms for water bottles.

#### 4.1.3. Haze Due to Additional Nucleation Sites

Preforms made with PCR may contain haze. Two likely reasons are the additional nucleation sites and the availability of smaller chains (that crystallize more readily), generating haze as a result. Often, preform haze can be rectified by increasing the cooling time in the injection cycle. Increases to cycle time have the negative impacts of reduced productivity, increased degradation, increased AA, and increased cost. The cycle time penalty can be anywhere between 10% and 30%.

#### 4.1.4. Degradation of IV

Intrinsic Viscosity (IV) is a gauge of molecular chain length and thus a predictor of strength of the material. When a resin goes through the injection molding process, it generally reduces in chain length. With virgin resins, for normal injection conditions and good drying practices, this loss is well understood and is usually no more than 0.03 dL/g. With PCR, this loss is relatively unpredictable. Mechanically and chemically recycled materials can have different residual compounds in the PCR that can negatively affect stability when processed through a high-heat and high-shear environment (e.g., the conditions inside of the extruder). Residual reactants and catalyst used to depolymerize PET during chemical recycling, if not removed effectively before PET is repolymerized, will act to depolymerize the PET during injection. The resulting drop in IV during injection could be significant. Sensitivity to degradation conditions may vary from batch to batch.

### 4.2. Preform and Container Appearance 

**Figure 13:**
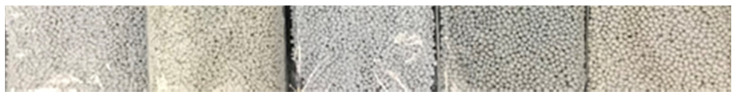
PCR resin color variation (image courtesy of Amcor).

The PCRs can vary significantly in color, between lots and between manufacturers. The variation between different grades is presented in Figure 13.

**Figure 14:**
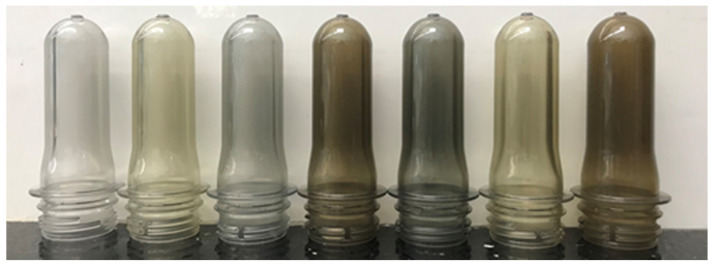
PCR preform color variation (image courtesy of Amcor).

The crystallinity in the pellet gives it a white appearance. This white envelope is not representative of the eventual color of a transparent article. An image of preforms made with 100% PCR from various manufacturers is shown in Figure 14. As can be observed in the image, appearance can vary significantly from manufacturer to manufacturer. 

**Figure 15:**
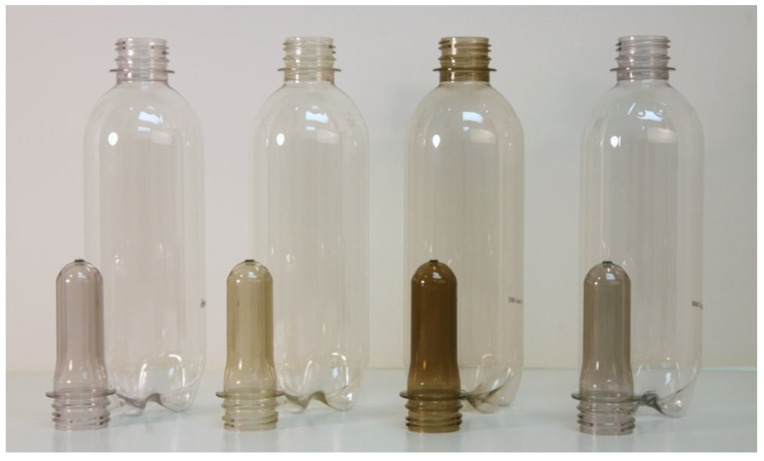
PCR preforms and bottles (image courtesy of Amcor).

As shown in Figure 15, a stark difference in color is seen at the preform level, where plastic wall thickness is about 10 times that of the final bottle. When preforms are converted into bottles, transparency increases, and color density decreases with the reduction of wall thickness. Color differences are most visible in the bottle finish, where thickness is maintained. 

Table 1 demonstrates the range of bottle mid-section color and haze, as measured on Colorquest™ equipment. With the thin wall, the lightness dimension of color scale (L* value) of the container is barely different than the virgin container. The other two dimensions a* (green-red dimension) and b* (blue-yellow dimension) are relatively closer. The difference in % haze and in yellowness index is more significant.

### 4.3. Blow Molding

#### 4.3.1. Preform Heating (Energy Requirements) 

Mobilizing the PET polymer chains for blow molding requires heating the PET in the preform body above its glass-transition temperature, most often accomplished with near-infrared radiation from heating lamps. Preform color affects the efficiency of absorbing near-IR radiation. Processing of the preform into a bottle can vary greatly when using PCR, as opposed to virgin PET. As discussed earlier, PCR composition varies. The amount of reheat additive can therefore vary from batch to batch. Material will vary in reheat concentration based on source. For example, if PCR is sourced from Asia, where reheat additives are not commonly used, the PCR will likely not have reheat additive. Similarly, for the American market, PET resins for water and CSD applications are likely to have lower levels of reheat additives than PET resins meant for heatset applications like isotonic drinks and juices. When US material is recycled, the resultant PCR will vary in reheat additive content from batch to batch based on changing concentrations of water, CSD, and heatset bottles. Generally, PCRs that appear cleaner are likely to require more heating energy (unless they have been supplemented with reheat additive) when blow molding, as compared to their darker PCR counterparts. 

Darker PCRs tend to have more gate swing or off-centered gates, leading to bottle wall thickness variation on the horizontal plane (also known as side-to-side wall thickness variation in the blow molding industry). Lot-to-lot preform color variation will impact IR absorption and the bottle material distribution. The process parameters (recipe) for blow molding may need to be adjusted every time bottles are produced. That said, those experienced in blow molding are able to adjust the processes whenever there is such a need. 

#### 4.3.2. Material Distribution Variation

Plastic bottle material distribution refers to the variation in weight in the vertical direction. In engineered applications, such as bottles meant for hot-fill or CSD, control over material distribution ensures the desired functional performance. As explained earlier, the process recipe may require finetuning with PCR composition and color changes. Polymeric chain lengths or molecular weights could have a wider distribution with PCR. As such, material stretch is more variable than for virgin PET, leading to more variation in the material distribution. These factors combined usually result in a more variable distribution of material within the body of the bottle, as compared to bottles made with virgin material. 

#### 4.3.3. Scrap Rate

Inclusions in the PCR PET can lead to container ruptures during blow molding. Variation in material distribution can lead to final container rejection. The scrap rate when producing containers from PCR PET tends to be higher than the scrap rate for virgin material. The scrap rate greatly depends on the specific grade and lot of PCR, the % PCR in the container, and the container performance requirements. A clean mechanically recycled or chemically recycled PET with few inclusions and high clarity would not be expected to have a significant scrap rate. Similarly, when blending 10% or 15% PCR with virgin PET, the scrap rate would not be expected to raise any red flags. Forgiving applications (such as non-pressurized water) and container shapes without complex geometry (or fine lettering) have wider specifications and wider operating windows, allowing operation at acceptable scrap rates with some container variation. Challenging and performance-demanding applications, such as hot fill and CSD, require precise material distribution. The scrap rate will tend to be higher for demanding applications. 

### 4.4. Container Performance 

#### 4.4.1. Bottle Shrinkage When Exposed to Heat

Dimensional stability is a concern for hot-fill containers, where bottles are exposed to temperatures equal to or exceeding 85 °C (185 °F) for many minutes. Issues with dimensional instability post-filling include shrinkage in height and/or diameter, uneven standing surfaces, and ovalization (impacting ability to properly secure a label). Virgin PET grades intended for hot-fill applications are designed to have lower levels of diethylene glycol (DEG). This particular byproduct of PET manufacturing leads to shrinkage in container height, diameter, and overall capacity, as well as deformation of features. For PCR, which is a mixture of different PET grades, the DEG content may vary drastically lot to lot. As a result, the shrinkage of the container post-hot-fill may vary. Shrinkage of bottles containing PCR would vary but would generally be expected to be higher than that observed in virgin-material containers. Careful bottle crystallinity management can mitigate this shrinkage. 

#### 4.4.2. UV Light Performance

**Figure 16:**
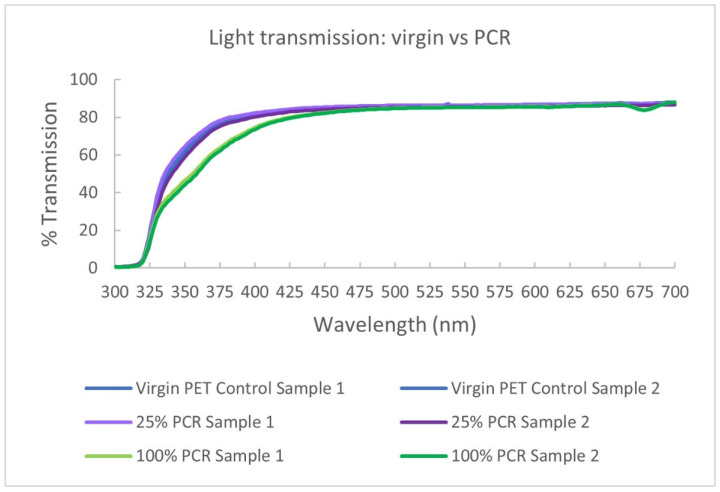
PCR impact on UV light transmission.

In some circumstances, PCR can reduce some UV light transmittance. As discussed in Section 4.2, containers made with PCR content may display a yellow/brown tint, especially with increasing content levels. Figure 16 is taken from one experiment where light transmission was measured on virgin, 25% PCR, and 100% PCR samples. In this specific case, the transmission of UV light (330 nm–400 nm) was reduced between 10% to 50% compared to the virgin control. This performance would again be affected by the grade and lot of PCRs. Food and beverage products with UV sensitivities may benefit from this attribute.

#### 4.4.3. Topload

Resistance to topload, also known as compressive strength, is the ability of the bottle to resist buckling under load. PET bottles can withstand a significant compressive load. As a result, secondary packaging for many beverage PET containers can be minimized for palletization. Introduction of PCR may impact the bottle’s compressive strength. If the PCR used is of lower average molecular weight than the virgin material it is replacing, increased PCR content may lead to decreased topload strength. Likewise, wide molecular weight distribution in bottles containing high PCR content may lead to variable topload performance.

#### 4.4.4. Burst Strength and Thermal Expansion

Burst strength and thermal expansion, which are critical performance attributes for pressurized containers (e.g., CSD products), may be affected by the lower average molecular weight and wider molecular weight distribution characteristic of some PCRs. Generally, burst pressures reduce, and expansions increase, with increasing PCR content. Inclusions in the plastic wall may lead to unpredictable and lower burst pressures. 

## 5. Application and Limitations

### 5.1. Cold-Fill Applications 

In many beverage applications, 100% PCR container options are available. These are mainly cold-fill beverage containers not exposed to pressure from carbonation or hot product. Such products include still water, chilled juices, tea, coffee, milk, and other cold-chained or aseptically filled (bottle sterilized with peracetic acid) products. The main functional requirements of these bottles are product containment, topload strength, and recloseability. These bottles are not required to withstand pressure, heat, or vacuum. 

**Figure 17:**
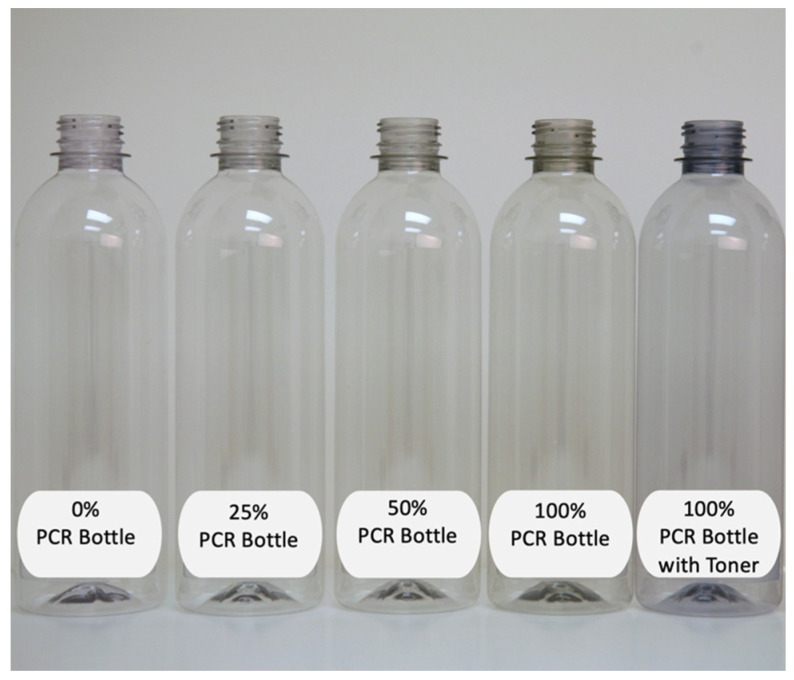
Impact on color with increasing PCR level (image courtesy of Amcor).

Color is a performance attribute impacting consumer visual appeal. One way to influence color is through the use of toners. Consumers tend to have a negative perception of slightly yellow containers for clear beverages like still water. Toners are typically designed to counterbalance yellow tones for such applications. A typical color range of containers containing PCR contrasted with one with toner is demonstrated in Figure 17.

Acetaldehyde may impact the flavor profile. When making bottles for flavorless products, like water, acetaldehyde in the PCR PET container needs to be monitored closely. AA scavengers may be used to mitigate risks associated with impacting the flavor profile. 

### 5.2. Heat-Set Bottles for Hot-Filled and Aseptic Products

Both hot-filled and some aseptic product containers require thermal stability. Aseptically filled containers that are sterilized with the hot application of hydrogen peroxide in the liquid or the vapor phase require thermal stability. Hot-filled containers are sterilized on contact with the heated product. As mentioned in Section 4.4.1, deformation on thermal treatment is minimized with adequate crystallinity. DEG content of the PCR will negatively impact the ability of the container to withstand heat without deformation. Containers that are filled hot also need to resist deformation resulting from vacuum generated when the product cools to room temperature or below. Material distribution within the container is critical to deformation resistance. 

Increased preform heating during blow molding will increase the final container crystallinity and the resistance of the container to thermal deformation. As preform temperature increases, material may drift off-center. Inability to achieve sufficient crystallinity may be a concern with Asian PCRs, without reheat additive. Both process and reheat additive use must be balanced to generate sufficient crystallinity. So far, there have not been any heat-set bottles with 100% PCR commercialized in the North American market. That should change soon.

### 5.3. Pressurized Containers

Containers used for pressurized products, such as carbonated soft drinks (CSD), present a significant challenge when increasing PCR content. While 10% or 15% PCR incorporation is not difficult, increasing to 100% PCR content is problematic. A very clean PCR grade is required. PET resins for CSD require longer molecular chain lengths (also known as higher intrinsic viscosity). Inclusions result in ruptures while blow molding, leading to holes in the container. Inclusions are most problematic when they occur in the highest stretch areas (e.g., the base feet). 

Bottles made with PCR may exhibit high expansion after filling with pressurized product. If not managed, expansion may lead to issues like label flagging and reduced shelf life. The single most important factor in forming 100% PCR bottles is the selection of the PCR. The PCR for CSD applications needs to be clean and of high molecular weight. Such PCRs are in limited supply. Some large beverage corporations have launched 100% PCR CSD bottles, but the roll-out has been limited due to supply limitations of the required PCR grades.

## 6. Regulatory Requirements

Plastic bottles are an attractive option for beverage packaging as they are recyclable and have a lower carbon footprint than alternative packaging. PCR PET must not pose a threat to consumer health and safety, especially for beverage packaging applications. During the collection process, non-food packaging containers and/or bottes containing additives that are not approved for food contact may enter the recycle stream. These materials are known as non-intentionally added substances (NIAS), substances that are not added to the food contact PET, but may be present in the bale and in the flake feed streams to the food grade PCR recycling process. These additives and materials will be present in the PCR PET and may contaminate food packaged within the final PCR PET article. It is important to quantify and control the risk, from a food safety perspective. Regulations regarding the reuse and recycling of PET vary between countries. In this section, we will cover some of the pertinent regulations related to PCR production and its use. This section will provide a framework for PCR’s acceptability in various countries. 

In the United States, the Food and Drug Administration (FDA) regulates the usage of PCR containers. There are three major concerns: (1) hazardous contaminant migration from the PCR to the food; (2) non-food-contact plastic and non-FDA-regulated material interaction with the packaged food, resulting from inclusions within the PCR; and (3) additives added to PCR that are not FDA compliant for food contact. To overcome these issues, the FDA considers the production of PCR on a case-by-case basis. The FDA issues informal notices on the suitability of PCR production processes for the production of food-contact-compliant material. Manufacturers considering the sale of PCR PET at a food contact grade must submit process documentation to the regulatory agency (FDA in the United States) for review. Three elements of the documentation are:

(1) Description of the controls that are in place to prevent non-PET plastics from entering the PCR production stream.
(2) Documentation and evidence of efficient contaminant removal. If requested, a surrogate contaminant will be used to validate the recycling process and demonstrate the effective removal of the contaminant. Additional migration modeling and testing can be used to demonstrate contaminant reduction to below 0.5 ppb (the dietary concentration that assumes negligible exposure for use with food products).
(3) Description of how the plastic will be used. With food contact materials, these descriptors include temperature range for use, type of food, duration of contact, and if the plastic will be used in a single-use or repeated application.

The FDA has determined that methanolysis and glycolysis tertiary recycling processes are suitable for the production of food-contact-grade PET; surrogate testing is not needed. Tertiary recycling processes are expected to produce high-purity materials. Mechanically recycled (secondary recycling) PCR PET does not allow for fine filtration and contaminant extraction. Hence, migration testing is necessary with mechanical recycling. Mechanically recycled PET must demonstrate residual reduction. Residual migration from the final PCR article must not exceed 1.5 μg/person/day estimated daily intake (EDI). There are several surrogates that are recommended for testing the efficacy of the recycling process. Challenge tests are designed for five categories of potential migrants: (1) volatile polar (chloroform, chlorobenzene, 1,1,1-Trichloroethane, diethyl ketone); (2) volatile non-polar (toluene); (3) heavy metal (copper, (II) 2-ethylhexanoate); (4) non-volatile polar (benzophenone, methyl salicylate); and (5) non-volatile non-polar (tetracosane, lindane, methyl stearate, phenylcyclohexane, 1-Phenyldecane, 2,4,6-Trichloroanisole). The surrogate test for heavy metals in PET is not required, based on evidence that PET does not readily absorb metal salts [116].

Regarding European Food Safety Authority (EFSA) regulation 282/2008, every recycling process needs to be approved before production [117]. While 5% of the feed stream to PET bottle recycling processes is assumed to be non-food containers on average, it could be up to 20% in specific instances [118]. The final contaminant concentration must be below 3 mg/kg (ppm) per substance. 

## 7. Conclusions and Future Trends

PCR PET production, conversion, and regulations are presented in this comprehensive review for the beverage industry. The push towards sustainability in beverage packaging would not be possible without the great efforts of material reclaimers, PET recyclers, converters, beverage manufacturers, and societal contributions from responsible consumers. Bottle-to-bottle recycling provides a significant reduction to environmental burdens.

In the last decade, the use of PCR PET has seen tremendous growth due to advancements in collection and recycling technology. Looking to the future of PET recycling, one of the most important areas of development is in the commercialization of chemical recycling technology, where PCR PET can be restored to virgin-like performance. Lab-scale technologies are rapidly scaling-up to pilot and production facilities. As an example of an advanced recycling, an enzyme-based technology has been developed to catalyze the hydrolysis of PET into TPA and EG [119]. In 2021, they announced the successful launch of their industrial demonstration plant, with a commercial manufacturing facility following by 2025.

**Figure 18:**
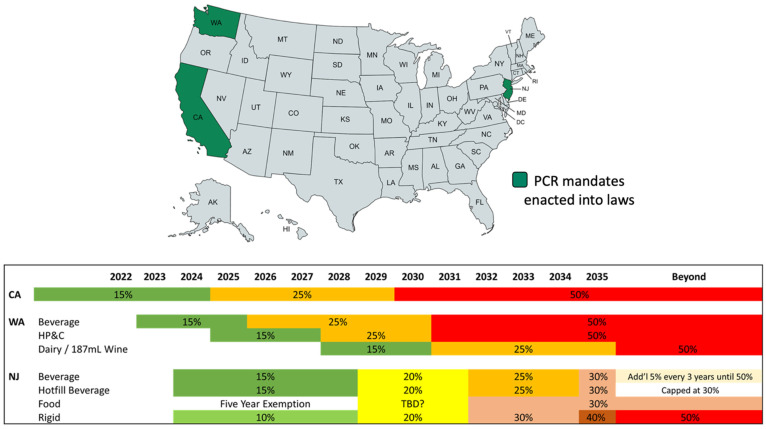
US PCR mandates across different states, California (CA), Washington (WA), and New Jersey (NJ), by May 2022.

There are several “speed bumps” to future increases in the conversion of PCR PET. Currently, there are only a handful of suppliers of PCR PET, and availability will be an issue as more beverage manufacturers adopt PCR PET. Limited supply will drive cost higher. As demand increases, PCR resin suppliers incur less pressure from competitors to provide a superior-quality product. Increased prices for PCR and variable quality are both barriers to adoption. Increasing public scrutiny of plastic packaging materials, from both environmental groups and legislative bodies, will spur adoption. Younger consumers are demanding the elevated use of recycled materials. Future investment in new recycling technologies and commercial adoption must be supported by recycling legislation and increased consumer recycling. The demand will continue to increase as PCR mandates come online. Mandates that have already been passed are depicted in Figure 18. California (CA), Washington (WA), and New Jersey (NJ) plan to implement up to 50% PCR beyond 2030–2035. Currently, there are no methods to detect the amount of PCR in a bottle, which presents a research opportunity that could be beneficial to recyclers and beverage manufacturers. This presents an interesting problem for the manufacturers and regulatory agencies, as enforcement of PCR mandates is difficult without such methods. In addition, there are also gaps in the detection of additives in PCR. Future research in detection methodologies will allow rapid prototyping and adoption of PCR in mainstream production and distribution of beverages.

The scientific data on combination of different grades of PET and how the mixture behaves in bottle manufacturing and performs for various applications is severely limited. The interaction of oxygen scavengers with PCR and its performance are also not available in the literature. Future research in this area can help with the adoption of PCR and the sustainability drive of beverage manufacturers.

Design enhancements of bottles for recycling (design for recycling) will be key to future collection and sortation to increase the yield and supply of PCR. The Association of Plastic Recyclers (APR) in the USA and the European PET Bottle Platform (EPBP) in the EU, both trade associations, have created a set of design guidelines for PET articles for recycling [120]. For example, the cap of the PET bottle can be made of either PP or PE. PP and PE are separable from the PET in the recycler’s float/sink tank. Polyesters classified with resin identification code (RIC) #1 must have a crystalline melting point between 225 °C and 255 °C.

Another initiative is digital watermarking for smart package recycling, also called HolyGrail 2.0. The idea of the initiative is to develop a technology for enhanced sortation of PET at a large scale [121,122,123]. The digital watermark could be the size of a postage stamp, integrated into the label and able to carry an array of information related to product, stock-keeping unit (SKU), manufacturer, type of plastic and layers, food vs. non-food use, and so on. Such design and traceability initiatives and technological breakthroughs may enhance the future of recycling.

Incorporation of PCR content into existing PET bottles is a first step towards a circular economy with zero leakage. Small steps will provide confidence in recycling processes, supplier’s capabilities, comparable cost, overall package, product performance, and eventually, wide adoption of the 100% PCR PET bottle.

## Figures and Tables

**Table 1:** Color and haze of different polyethylene terephthalate (PET) bottles made with different post-consumer recycled (PCR) grades.

PET Type	L*	a*	b*	% Haze	Yellowness Index
Virgin	94.61	−0.04	0.60	0.74	1.13
PCR Grade A	94.83	−0.10	0.77	2.34	1.39
PCR Grade B	94.88	−0.14	1.32	1.92	2.42
PCR Grade C	93.19	−0.17	2.64	6.20	4.99
PCR Grade D	94.65	−0.14	0.82	2.59	1.47

L* = 100 bright/0 dark, a* = +red/−green, and b* = +yellow/−blue.
